# Supplementary figures and images for: The microRNA-mediated gene regulatory network in the hippocampus and hypothalamus of the aging mouse
Source: PLoS One. 2023 Nov 9;18(11):e0291943. doi: 10.1371/journal.pone.0291943 (PMC10635555; doi:10.1371/journal.pone.0291943)

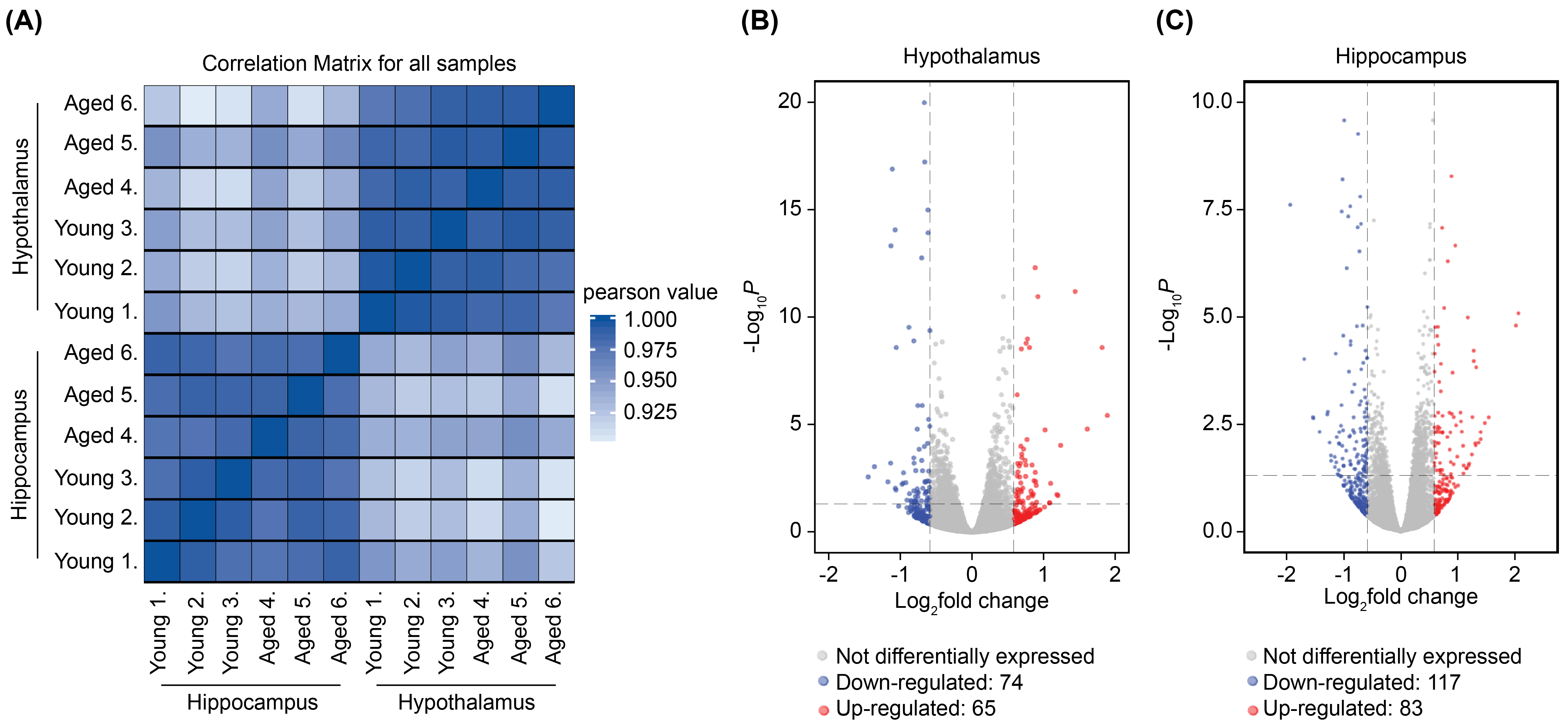

Supplement: S1 Fig — (A). Heatmap of Pearson’s correlation coefficients for gene expression in each young and aged mouse brain hypothalamus and hippocampus sample. The X and Y axes represent each sample, and the color represents the correlation coefficient. (B, C). Volcano plots of all detected genes from mRNA sequencing analysis of young versus aged hypothalamic and hippocampal tissues. Differentially upregulated genes are colored red, differentially downregulated genes are colored blue, and non-significant genes are colored gray. The cut-off was set as fold change > 1.5, and statistical significance was set as padj < 0.05. (TIF) [file pone.0291943.s001.tif]

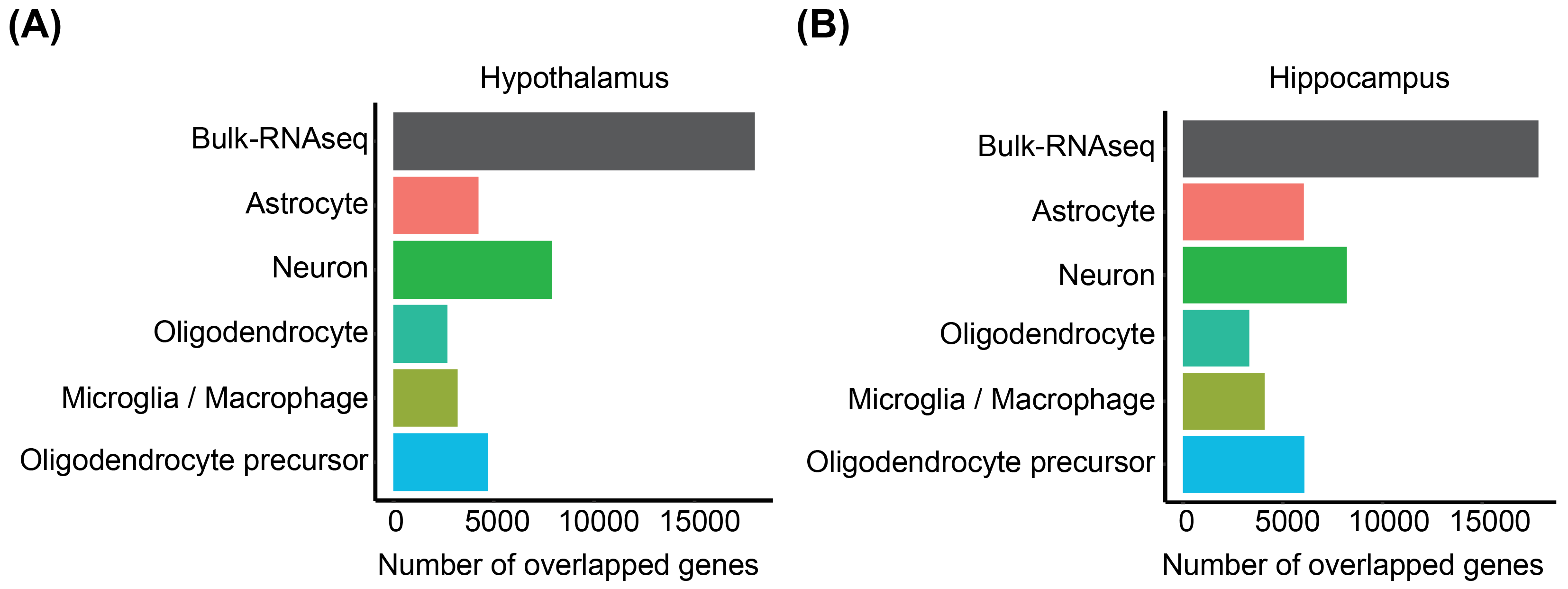

Supplement: S2 Fig — (A) Bar graph showing the number of gene distributions covered for each cell type in hypothalamic tissue in bulk RNA sequencing versus single nucleus RNA sequencing datasets. (B) Bar graph showing the number of gene distributions covered for each cell type in hippocampal tissue in bulk RNA sequencing versus single nucleus RNA sequencing datasets. (TIF) [file pone.0291943.s002.tif]

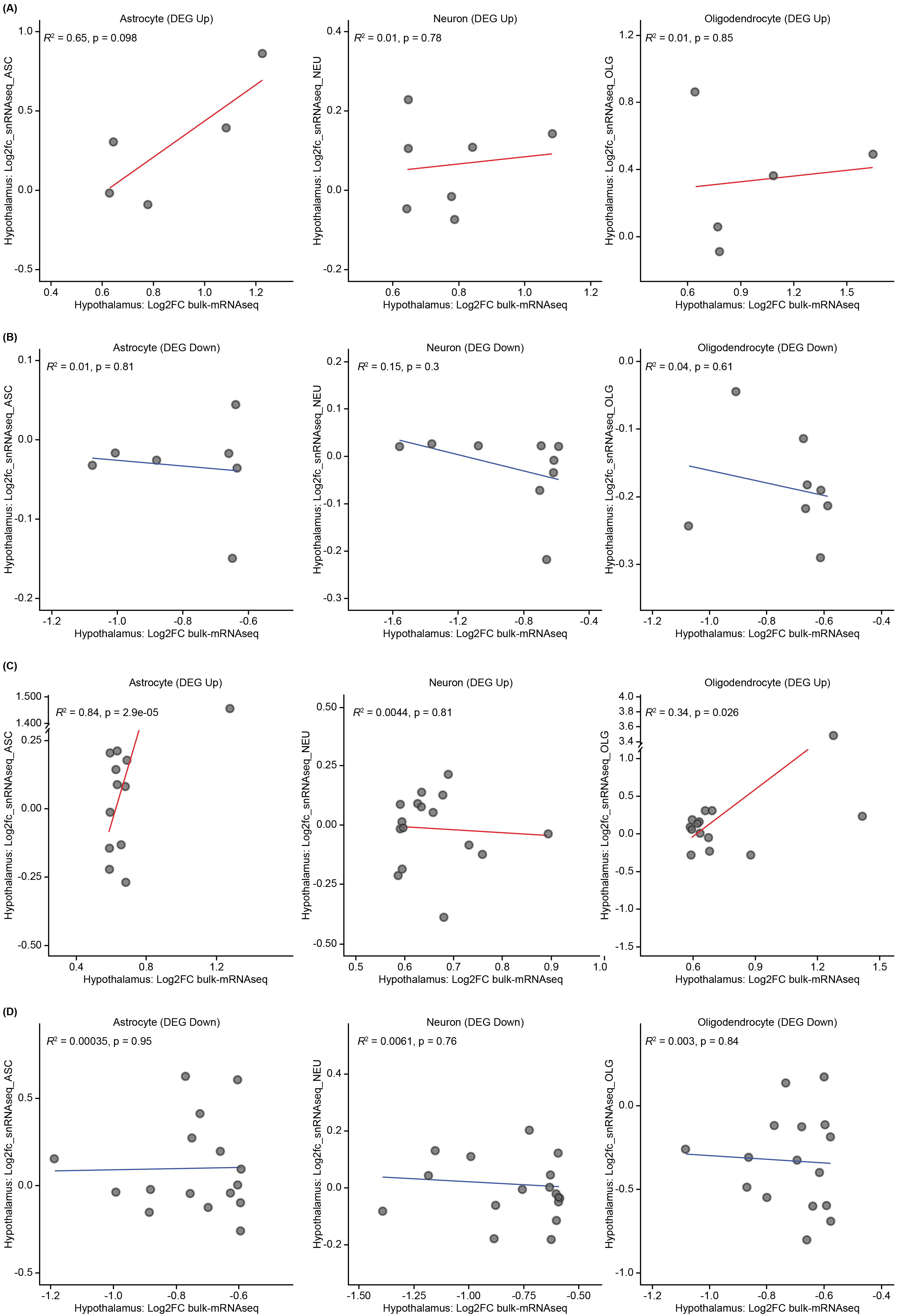

Supplement: S3 Fig — (A). Pearson’s correlation analysis of Log2FC expression of differentially upregulated genes in bulk mRNA sequencing (cut-off value: fold change > 1.5, padj < 0.05) and single nucleus RNA sequencing (cut-off value: log2FC > 0.1, padj < 0.05) datasets from hypothalamic tissue. Linear regression is indicated with the colored line. The Pearson’s correlation coefficient (R2) and p-value are displayed in the graph. Gene expression changes in hypothalamic astrocytes (R2 = 0.65, p = 0.098), oligodendrocytes (R2 = 0.01, p = 0.85) and neurons (R2 = 0.01, p = 0.78) are not correlated. (B) Pearson’s correlation analysis of Log2FC expression of differentially downregulated genes in bulk mRNA sequencing (cut-off value: fold change > 1.5, padj < 0.05) and single nucleus RNA sequencing (cut-off value: log2FC > 0.1, padj < 0.05) datasets from hypothalamic tissue. Linear regression is indicated with the colored line. The Pearson’s correlation coefficient (R2) and p-value are displayed in the graph. Gene expression changes in hypothalamic astrocytes (R2 = 0.01, p = 0.81), oligodendrocytes (R2 = 0.04, p = 0.61) and neurons (R2 = 0.15, p = 0.3) are not correlated. (C) Pearson’s correlation analysis of Log2FC expression of differentially upregulated genes in bulk mRNA sequencing (cut-off value: fold change > 1.5, padj < 0.05) and single nucleus RNA sequencing (cut-off value: log2FC > 0.1, padj < 0.05) datasets from hippocampal tissue. Linear regression is indicated with the colored line. The Pearson’s correlation coefficient (R2) and p-value are displayed in the graph. Gene expression changes in hippocampal astrocytes (R2 = 0.84, p = 2.9e-05) and oligodendrocytes (R2 = 0.34, p = 0.029) are significantly correlated, whereas gene expression changes in hippocampal neurons (R2 = 0.0044, p = 0.81) are not correlated. (D) Pearson’s correlation analysis of Log2FC expression of differentially downregulated genes in bulk mRNA sequencing (cut-off value: fold change > 1.5, padj < 0.05) and single [file pone.0291943.s003.tif]

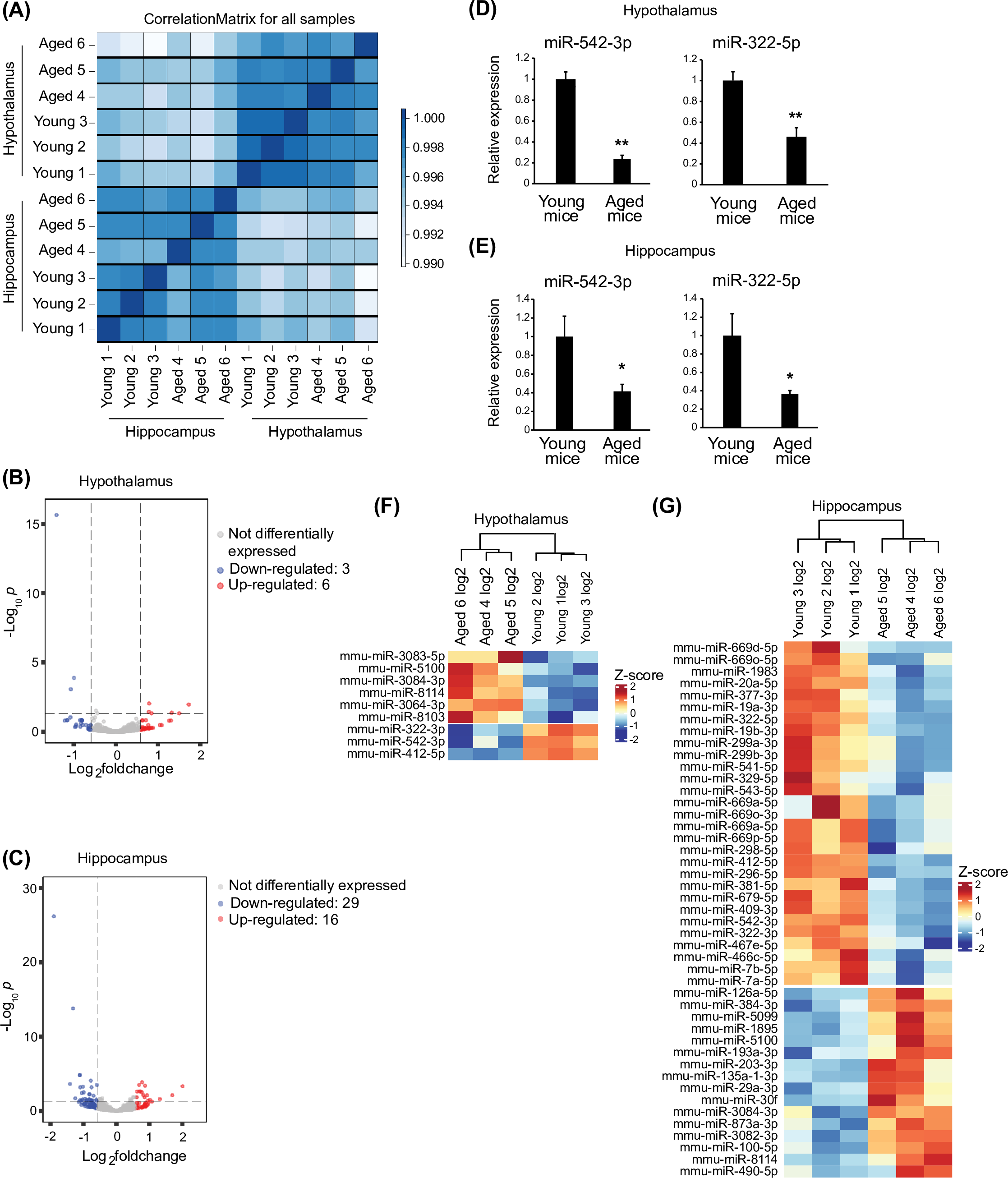

Supplement: S4 Fig — (A) Heatmaps of Pearson’s correlation coefficients of log2(Count+1) values for miRNA expression of each young and aged mouse brain from hypothalamic and hippocampal tissue samples. The X and Y axes represent each sample, and the color represents the correlation coefficient. (B, C) Volcano plots of detected miRNA expression from miRNA sequencing datasets of young and aged mouse brain from (B) hypothalamic and (C) hippocampal tissues. Differentially expressed genes (cutoff value: fold change > 1.5, padj < 0.05) are colored red for upregulated genes, blue for downregulated genes, and gray for non-significant genes. (D, E) Validation of miRNA expression from small-RNA sequencing datasets by qRT-PCR in the (D) hypothalamic and (E) hippocampal tissues (n = 4). Data are presented as mean ± SEM. **p < 0.01, *p < 0.05. (F, G) Heatmap of differentially expressed miRNAs (cut-off value: fold change > 1.5, padj < 0.05) count numbers (TPM log2) in young and aged mouse brain samples from (F) hypothalamic and (G) hippocampal tissues. (TIF) [file pone.0291943.s004.tif]

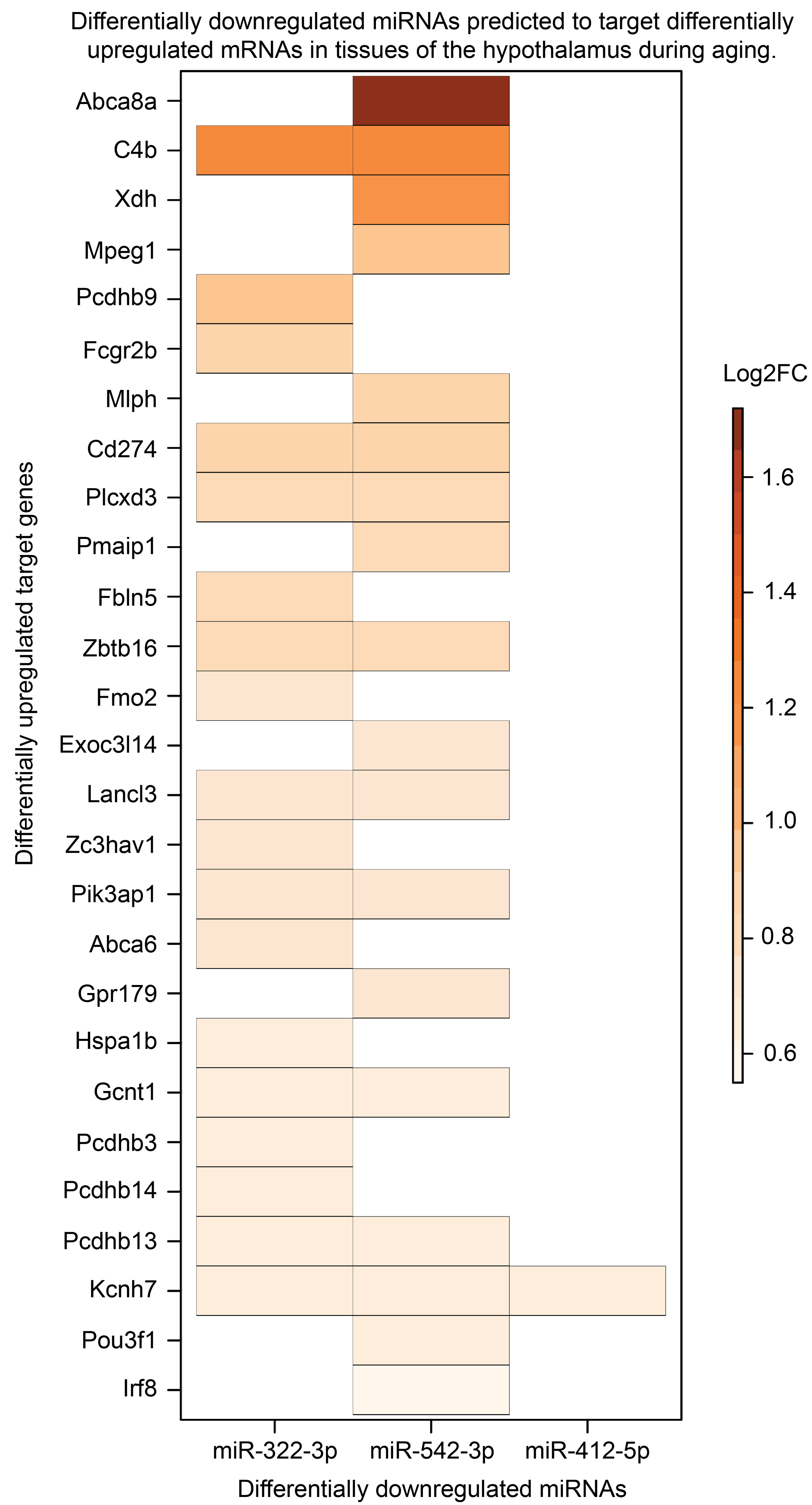

Supplement: S5 Fig — Heatmap shows differentially upregulated gene expression (cut-off value: fold change > 1.5, padj < 0.05) predicted to be targeted by differentially downregulated miRNAs (cut off value: fold change > 1.5, padj < 0.05) in mRNA sequencing and small-RNA sequencing datasets from hypothalamic tissue. (TIF) [file pone.0291943.s005.tif]

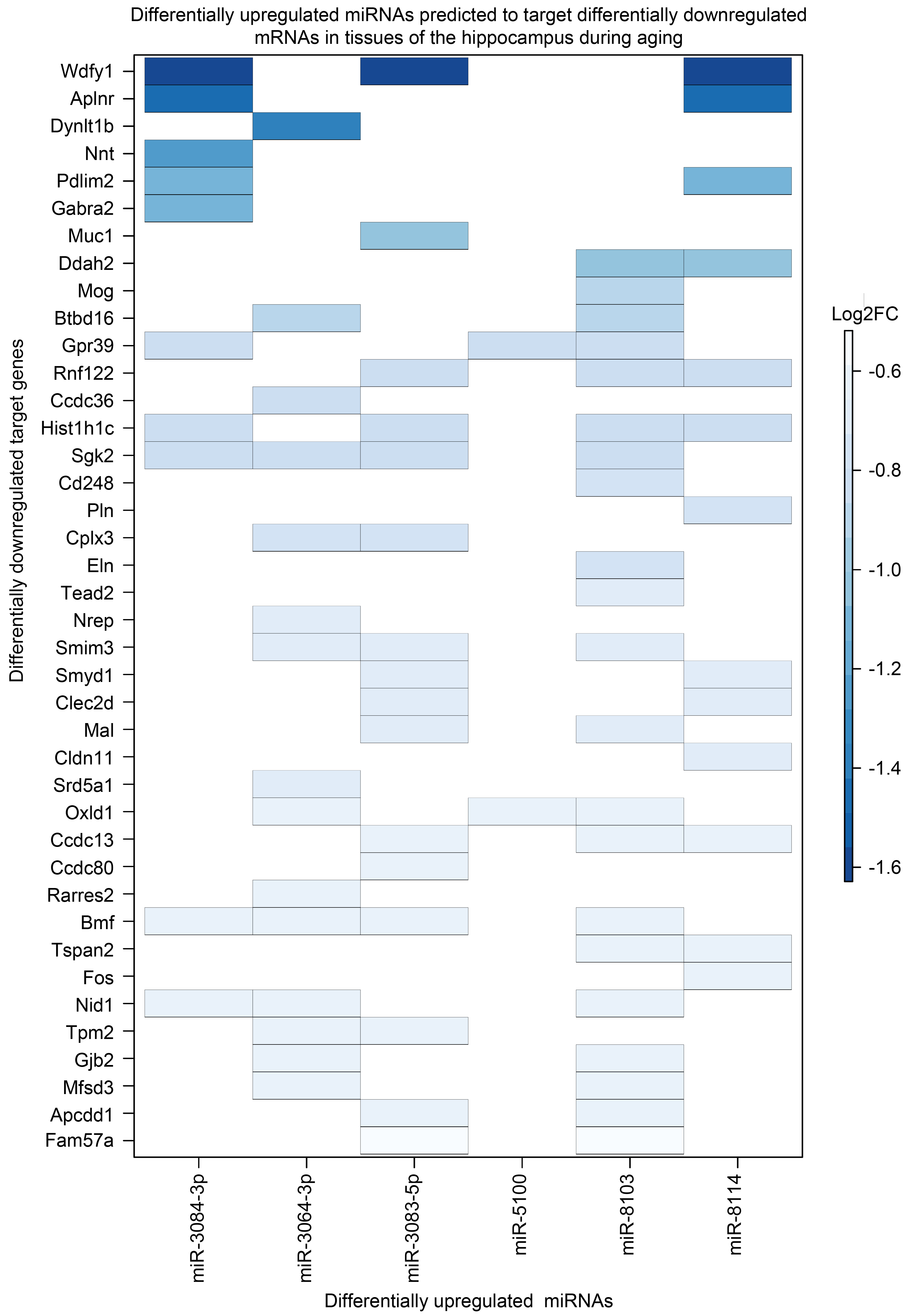

Supplement: S6 Fig — Heatmap shows differentially downregulated gene expression (cut-off value: fold change > -1.5, padj < 0.05) predicted to be targeted by differentially upregulated miRNAs (cut off value: fold change > 1.5, padj < 0.05) in mRNA sequencing and small-RNA sequencing datasets from hypothalamic tissue. (TIF) [file pone.0291943.s006.tif]

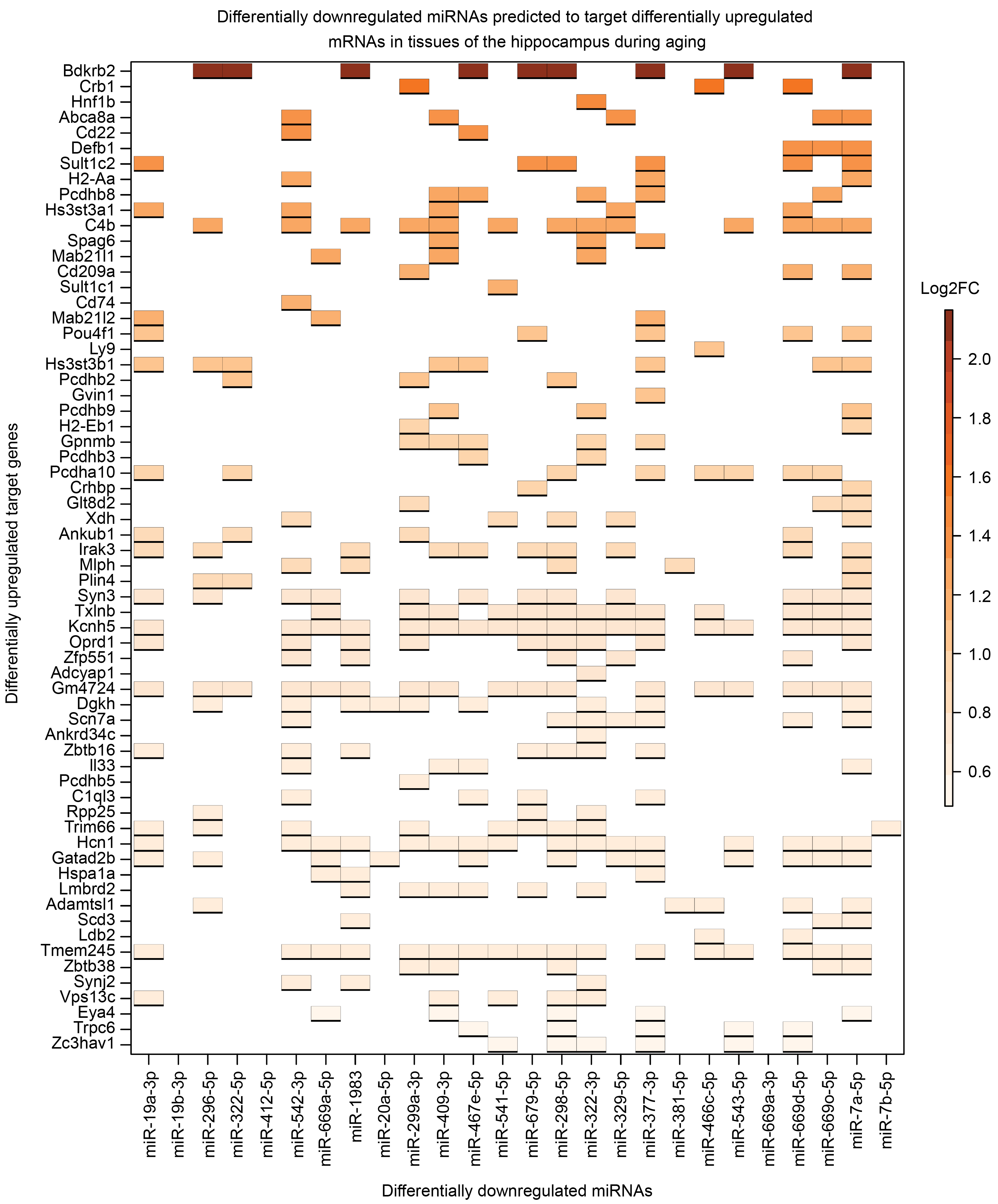

Supplement: S7 Fig — Heatmap shows differentially upregulated gene expression (cut-off value: fold change > 1.5, padj < 0.05) predicted to be targeted by differentially downregulated miRNAs (cut-off value: fold change > 1.5, padj < 0.05) in mRNA sequencing and small-RNA sequencing datasets from hippocampal tissue. (TIF) [file pone.0291943.s007.tif]

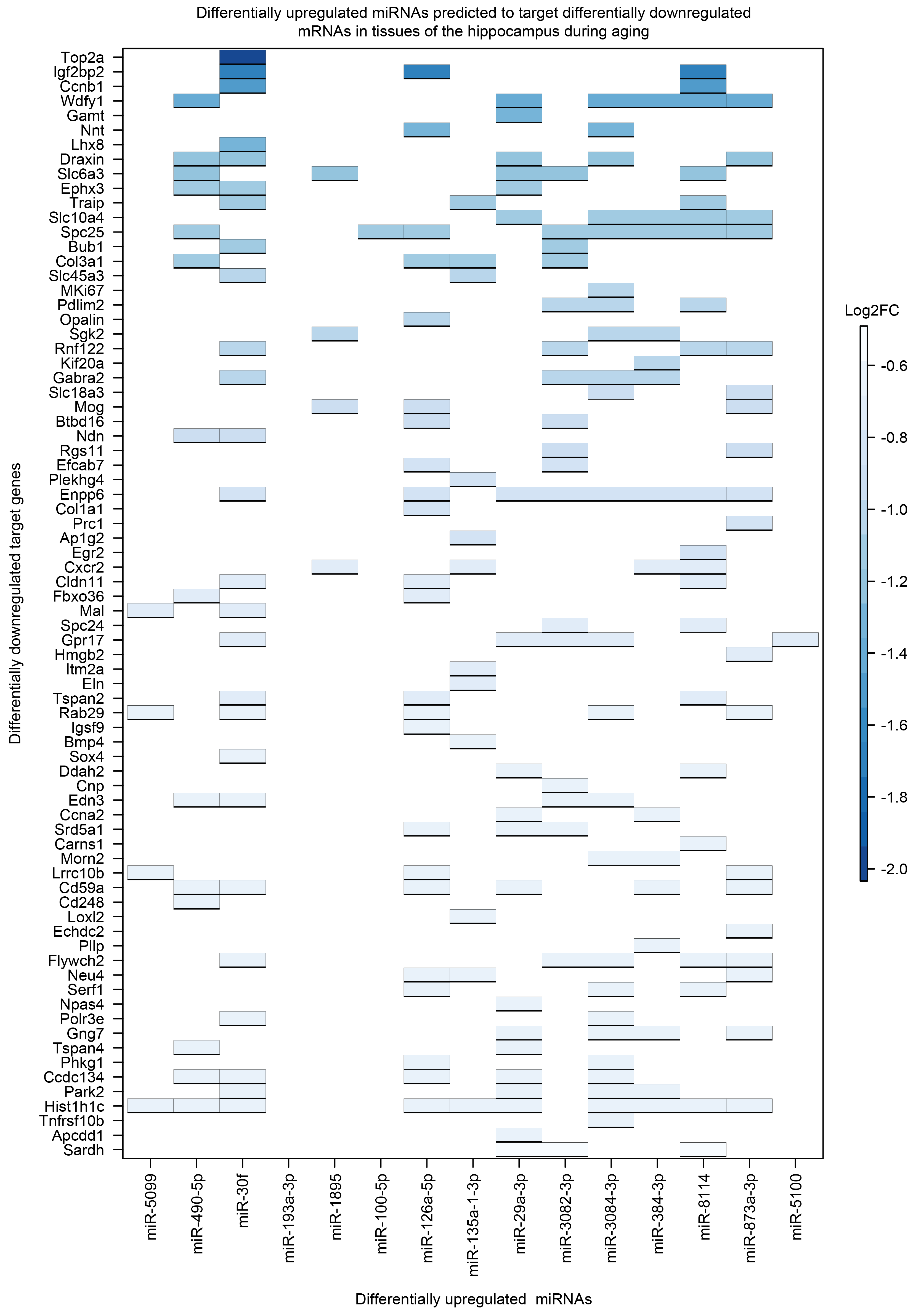

Supplement: S8 Fig — Heatmap shows differentially downregulated gene expression (cut-off value: fold change > -1.5, padj < 0.05) predicted to be targeted by differentially upregulated miRNAs (cut-off value: fold change > 1.5, padj < 0.05) in mRNA sequencing and small-RNA sequencing datasets from hippocampal tissue. (TIF) [file pone.0291943.s008.tif]

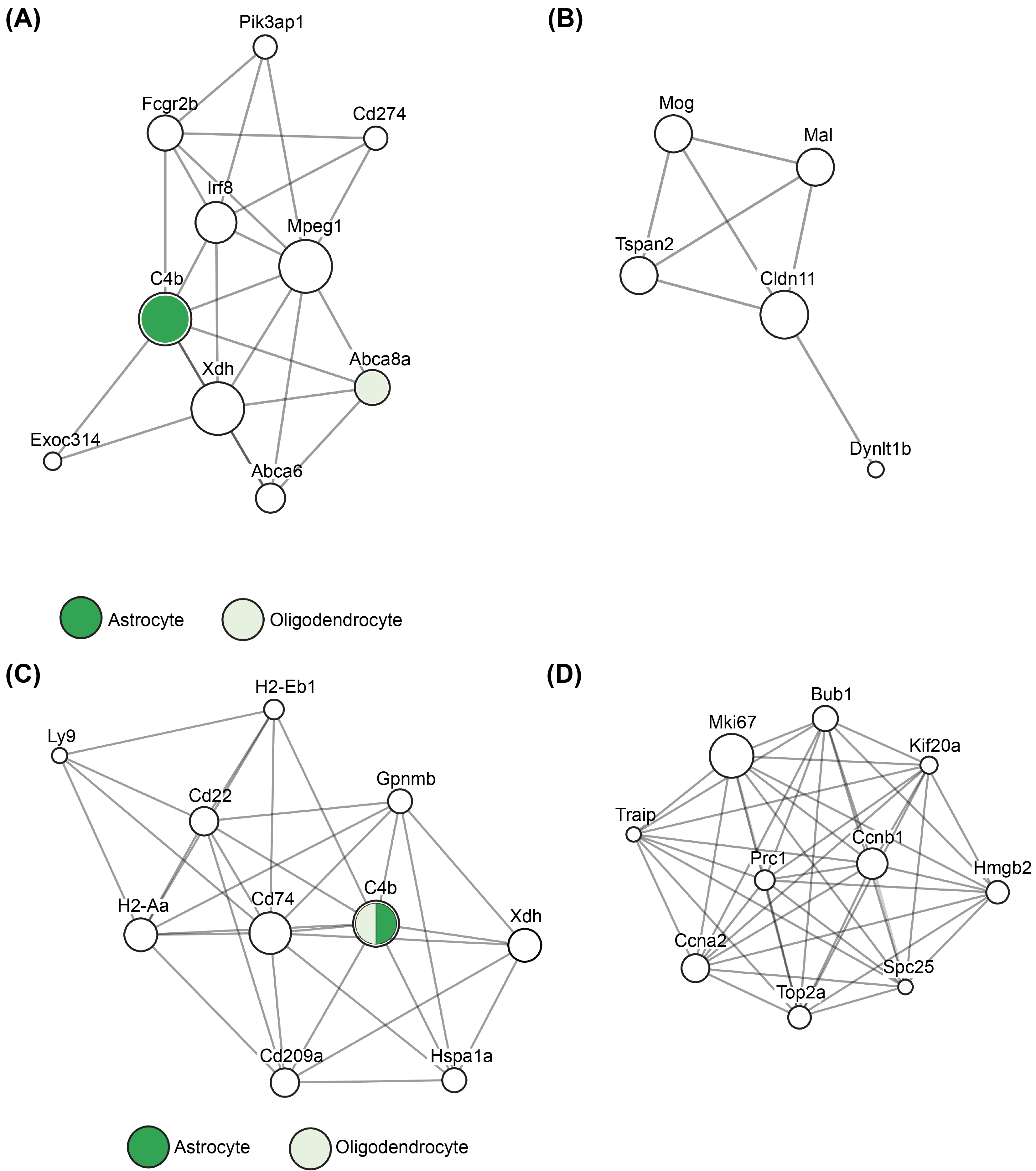

Supplement: S9 Fig — (A) PPI analysis of differentially upregulated genes targeted by differentially downregulated miRNAs in hypothalamic tissues, derived from mRNA sequencing datasets and small-RNA sequencing datasets. PPI network analyzed by STRING and MCODE, node sizes for target genes are determined by the degree method, and node color indicates the cell type. (B) PPI analysis of differentially downregulated genes targeted by differentially upregulated miRNAs in hypothalamic tissues, derived from mRNA sequencing datasets and small-RNA sequencing datasets. PPI network analyzed by STRING and MCODE, node sizes for target genes are determined by the degree method, and node color indicates the cell type. (C) PPI analysis of differentially upregulated genes targeted by differentially downregulated miRNAs in hippocampal tissues, derived from mRNA sequencing datasets and small-RNA sequencing datasets. PPI network analyzed by STRING and MCODE, node sizes for target genes are determined by the degree method, and node color indicates the cell type. (D) PPI analysis of differentially downregulated genes targeted by differentially upregulated miRNAs in hippocampal tissues, derived from mRNA sequencing datasets and small-RNA sequencing datasets. PPI network analyzed by STRING and MCODE, node sizes for target genes are determined by the degree method, and node color indicates the cell type. (TIF) [file pone.0291943.s009.tif]

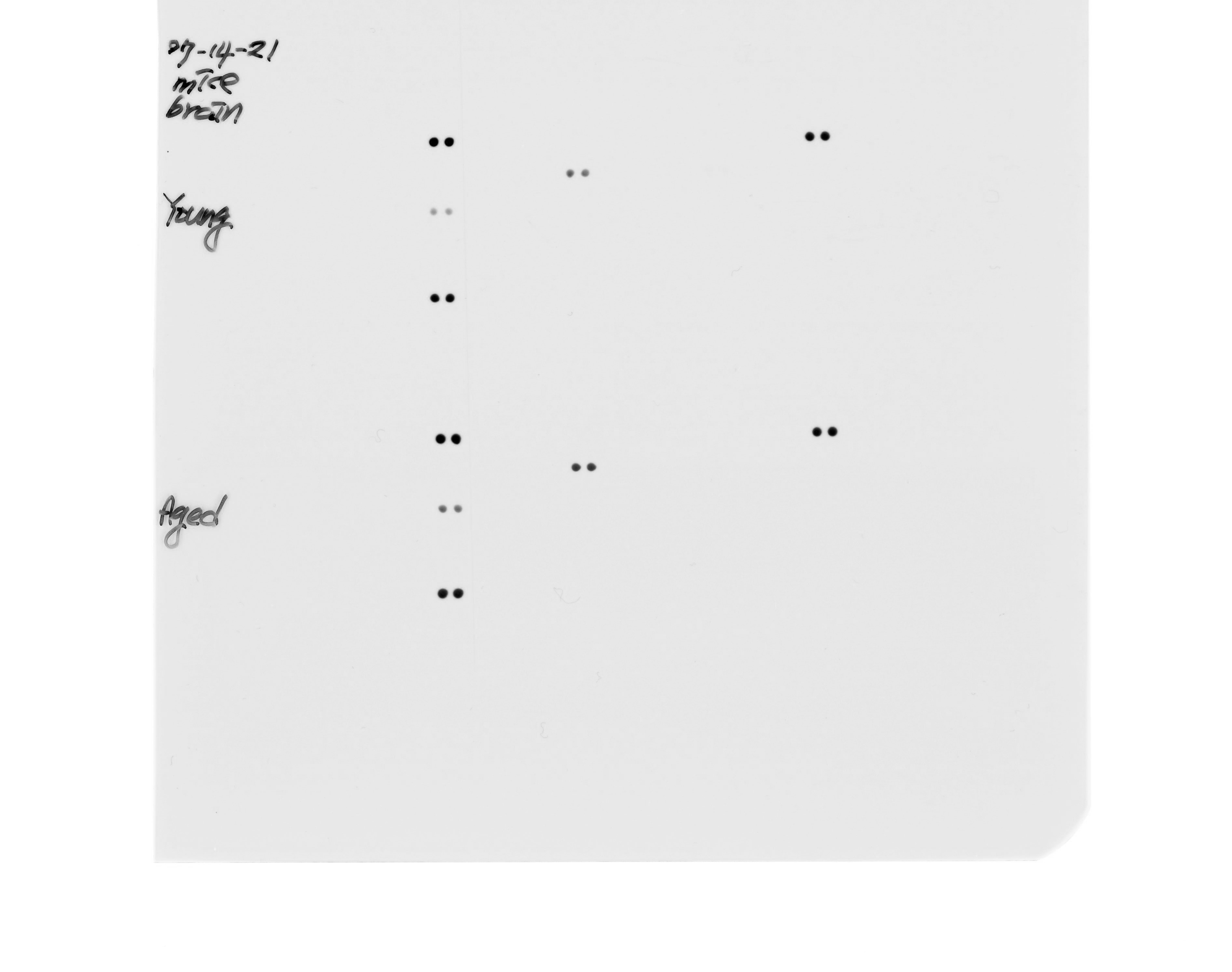

Supplement: S1 Dataset — (TIF) [file pone.0291943.s012.tif]
