# Supplementary material for: The microRNA-mediated gene regulatory network in the hippocampus and hypothalamus of the aging mouse
Source: PLoS One. 2023 Nov 9;18(11):e0291943. doi: 10.1371/journal.pone.0291943 (PMC10635555; doi:10.1371/journal.pone.0291943)
Supplement: Attachment — Submitted filename: Response_to_Reviewers_PloSoNe_v6.docx [file pone.0291943.s014.docx]

**Response to the Reviewer’s comments**

We would like to thank the reviewers for their constructive comments and helpful suggestions. We hope that our responses are clear and concise.

**Reviewer #1**

[1. The](http://1.the/) text, especially the introduction part, could be more concise to help the reader. I would suggest the introduction to be more focused. Avoid citing several, in most cases very old references, that obscure the reader. Please carefully, revise the references, as in several cases they are not cited in the most appropriate position in the manuscript, and reduce the references cited by keeping the most relevant ones.

We appreciate the thorough and constructive comment. As suggested by the reviewer, we revised the references, reducing and removing several old references and only included the most relevant ones in the introduction.

2. As the authors have stated, their RNA-seq and small RNA-seq analysis identified a relatively low number of differentially expressed genes and miRNAs compared to similar studies. It would be nice if they provide an explanation of possible reasons, that could be for example related with the differential expression analysis packages they used. DeSeq2, used for the miRNA analysis in this study, is commonly used for differential expression analysis. In the results section, instead of commenting on selected transcripts (Cd22, Irak3), it would be better to compare their findings with previous similar studies (for example ref 10 and https://doi.org/10.1016/j.gpb.2020.12.001 for mRNA analyses) and provide a schematic representation (e.g. Venn diagrams) of the detected overlaps.

As suggested by the reviewer, we conducted a comparative analysis of differentially expressed genes (DEGs) between our study and two previous investigations by Li et al. [1], and Srivastava et al. [2]. Li et al. focused on the hippocampus tissues of male mice at different ages (2, 12, and 24 months). Their RNA-seq analysis encompassed trimming, mapping, read count quantification and differential expression assessment through CLCbio software (CLC Genomics Workbench 7.0.2, CLC genomics Server). However, they did not provide the processed data of mapped read counts (SRP271007), impeding our ability to evaluate discrepancies stemming from the “differential expression analysis” tools. On the other hand, Srivastava et al. provided the processed RNA-seq data from whole brain tissue of 2- and 15-month-old mice (GSE75192). Our own DESeq2 analysis unveiled 52 downregulated and 44 upregulated genes, aligning closely with the quantities of DEGs we identified in our RNA-seq data. Nonetheless, we were unable to confirm the possibility that different algorithms may impact the generation of DEGs through this analysis.

Additionally, we compared the DEGs from the three studies. Li et al found 229 upregulated and 86 downregulated in both 12- and 24- months old mice when compared to the 2-month-old mice. However, they only provided a list of cognitive dysfunction-related differentially expressed genes, instead of providing the full list of differentially expressed genes. Hence, we compared our DEGs with their cognitive dysfunction-related DEGs (72 upregulated and 53 downregulated) in the hippocampus of 12-month-old mice versus 2-month-old mice, representing early aging stages. Fig. R1 indicates that there were no overlapping upregulated genes between our RNA-seq data and previous similar studies. However, for downregulated genes, we found three overlapping genes (Defb1, Spag6, Ly9) between our RNA-seq data and a study by Li et al. These overlapping transcripts (Defb1, Spag6, Ly9) were included in the results section (page 6).

**Fig. R1.** Venn diagram shows overlapping (A) differentially upregulated and (B) differentially downregulated genes reported by previous studies of Li et al. and Srivastava et al. and our study. (a) represents differentially expressed genes (cut-off value: fold change > 1.5, padj < 0.05) of young (2 months) and aged (15 months) male mouse whole brain tissue reported by Srivastava et al. (b) and (c) represent differentially expressed genes (cut-off value: fold change > 1.5, padj < 0.05) of our RNA-seq data derived from (b) hippocampal and (c) hypothalamic tissue of young and aged male mice. (d) represents differentially expressed genes (cut-off value: Log_2_FC > 1.0, p < 0.05) of young (2 months) and aged (12 months) male mouse hippocampal tissue reported by Li et al.

3. The authors highlighted C4b upregulation during aging, which has also been reported in neurodegenerative and neurological disorders, such as Alzheimer’s and Schizophrenia. To my opinion it would be interesting to include in the discussion a kind of parallelism of this finding between aging and neurodegenerative / neurological disorders.

As suggested by the reviewer, we included previous studies which emphasized upregulation of C4b in complement and coagulation pathways in neurodegenerative/neurological disorders in the discussion section (page 19). We also pointed out the significance of studying miR-542-3p, miR-322-3p and C4b regulatory networks in models of neurodegenerative/neurological disorders.

4. Regarding materials and methods: Please provide details on the miRNA mimics used for the transfections and state at which point following transfection the cells were harvested for further analysis. Please provide the primer sequences used for C4b qRT-PCR and comment on the selection of the housekeeping gene used. Also, provide a description either in the materials and methods or in the corresponding figure legend (Fig. 2) of the module score.

We have listed the primer sequences for the C4b and housekeeping 36B4 genes that we used for the RT-PCR experiments in the Materials and Methods section. As suggested by the reviewer, we also mentioned the module score in the appropriate figure legends (Figs. 2C and 2D) and Materials and Method section. We also provided the information regarding the miRNA mimics used for transfection, as well as detailed procedures for cell harvest (page 21).

5. Regarding figures: Figure 4: E the statistics symbol is missing. If there is no statistical significance in the result, please note it in the legend. C: include the statistic symbols (*, **) meaning to the legend. Figure S4: D: missing the graph for miR-322-5p validation E: please check spelling. The figure shows miR-522-3p. Also, the statistics symbols are missing from E. If no statistical significance, please note it in the legend. Include the statistic symbols (**) meaning to the legend.

Thank you for bringing the error to our attention. We added the statistics symbol which was missing in Fig. 4E. For Figs. S4D and S4E, we checked the data again and presented both miR-542-3p and miR-322-5p validation results. The statistics symbols (*, **) were defined in the legend.

**Reviewer #2**

1. The manuscript provides a comprehensive and meticulous analysis of age-related changes in gene expression and microRNA (miRNA) profiles in the hypothalamus and hippocampus regions of the mouse brain. The authors employed a robust experimental design using both young and aged mouse groups, coupled with both mRNA and small-RNA sequencing. The use of well-established bioinformatics tools like HISAT, StringTie, and Bowtie2 ensured accurate mapping, prediction, and comparison of novel genes and transcripts. Their rigorous approach to data normalization and determination of differentially expressed genes is commendable.

Thank you for the positive comment.

2. However, the study has some limitations. First, the study used only male mice, limiting the generalizability of the results to female mice or other species. Sex-specific differences in gene expression could impact the aging process, so a more comprehensive study including both sexes might provide a broader perspective. Secondly, while the authors have measured a substantial number of variables, it might have been beneficial to have more biological replicates in each group to increase statistical power and the reliability of the findings.

We appreciate the reviewer’s comment. Our RNA-seq data were generated from only male mice and we could not find comparable RNA-seq data from female mice. Therefore, within the limited time, we evaluated several differentially expressed genes in the hippocampal region of age-matched (2- and 15-month-old) female mice (Fig. R2).

**Fig. R2. Validation of differentially expressed genes in the hippocampal tissue by qRT-PCR during aging of the female mouse brain.** (A) Upregulated genes in hippocampal tissue. (B) Downregulated genes in hippocampal tissue. Five differentially expressed genes (cut-off value: fold change > 1.5, padj < 0.05) from RNA-seq data of male hippocampal tissues, were examined. Data are presented as mean ± SEM. **p < 0.01, *p < 0.05.

We performed qRT-PCR to evaluate several differentially expressed genes in female mice, as identified from RNA-seq data of male hippocampal tissues. Many of these differentially expressed genes showed similar tendencies in both male and female mice, highlighting potential common mechanisms during brain aging. However, to fully understand both shared and sex-specific biological processes, further analysis is required, which has been mentioned in the discussion section (page 17).

3. Furthermore, while the authors have used in vitro studies using astrocyte cell lines, integrating data from a wider range of neuronal and non-neuronal cell types could be beneficial, given the cellular diversity of the brain and the potential influence of other cell types on aging processes.

As suggested by the reviewer, we also used non-neuronal microglial cell line to validate target gene expression following mimic miR-322-3p and miR-542-3p transfection. However, C4b target gene expression in the microglial cell line was not changed after mimic miR-322-3p and miR-542-3p transfection (Fig. R3).

**Fig. R3.** **Target gene expression analysis in the microglial cell line following mimic miR-322-3p and miR-542-3p transfection using qRT-PCR.** Data are expressed as mean ± SEM. **p < 0.01, * p < 0.05, * ns (not significant) by Student’s two-tailed t-test.

4. Overall, while the manuscript is robust in its analysis and provides valuable insights into the aging process at the molecular level, further research, including both sexes and a wider array of cell types, could be beneficial to present a more comprehensive picture of age-related changes in the brain.

We appreciate the reviewer’s comments and agree that inclusion of both sexes and a wider array of cell types will allow more comprehensive elucidation of age-related changes in the brain. Therefore, in further studies, we plan to isolate hippocampal, hypothalamic, cortex and cerebellum brain tissues of both sexes using a wide range of young and aged mice to simultaneously perform bulk RNA-seq and small-RNA sequencing.

5. Moreover, the authors' representation of the current literature appears incomplete and could be greatly enriched by referencing recent relevant work in this field, particularly studies focusing on the aging brain in other species. A recent gene-expression meta-analysis (doi: 10.3389/fnins.2022.915907) explored the changes from multiple gene expression studies of the hippocampus in aging rats from a molecular interaction perspective and found substantial alterations related to immune functions and immunoglobulin dynamics. This lends credence to the concept of inter-species commonalities in brain aging processes and should be mentioned in the Introduction and / or the Discussion. It is essential for the authors to contextualize their study within the broader framework of aging research.

Thank you for drawing our attention to this reference required for our discussion. As suggested by the reviewer, we included recent work by Giannos and Prokopidis [3] (doi: 10.3389/fnins.2022.915907), which involved meta-analysis of publicly available gene expression data of hippocampal tissues of young (5–8 months) and aged (21–26 months) male rats to identify clustered protein-protein interaction networks of differentially expressed genes, in the discussion section (page 19). Similar to our result, their highly-ranked single cluster of 19 age-related hippocampal differentially expressed genes was also associated with immune response.

6. From a proofreading perspective, the manuscript is generally well-written and organized. The language is clear, concise, and uses standard scientific terminology correctly. The methods section follows a logical progression, detailing the steps of the analysis sequentially and making it easier to follow.

Thank you for the positive comments on our manuscript.

7. However, there are a few areas that could be improved for clarity and precision. The authors should consider using a more consistent style when referring to various software packages, databases, and resources. For instance, in some cases, URLs are provided, while in others, they are not. This could potentially cause confusion for readers trying to replicate the study. Also, the manuscript often presents several methods sequentially without much transition or explanation of why each step is necessary. Providing more context or rationale for each step of the method might improve clarity for readers unfamiliar with the specific techniques used. Lastly, sentences are quite long and packed with information. Breaking these up into smaller, more digestible sentences might enhance readability. For instance, the sentence starting with "For small-RNA sequencing..." could be split into two for clarity.

Thank you for your suggestion. As suggested by the reviewer, we included URLs for the software packages, databases, and resources used in this study. In the Methods section, we explained each step more clearly, and enhanced readability by shortening the sentences.

**References**

1.	Li M, Su S, Cai W, Cao J, Miao X, Zang W, et al. Differentially Expressed Genes in the Brain of Aging Mice With Cognitive Alteration and Depression- and Anxiety-Like Behaviors. Front Cell Dev Biol. 2020;8: 814. doi:10.3389/fcell.2020.00814

2.	Srivastava A, Barth E, Ermolaeva MA, Guenther M, Frahm C, Marz M, et al. Tissue-specific Gene Expression Changes Are Associated with Aging in Mice. Genomics, Proteomics & Bioinformatics. 2020;18: 430–442. doi:10.1016/j.gpb.2020.12.001

3.	Giannos P, Prokopidis K. Gene Expression Profiles of the Aging Rat Hippocampus Imply Altered Immunoglobulin Dynamics. Front Neurosci. 2022;16: 915907. doi:10.3389/fnins.2022.915907
